# Sequential delithiation behavior and structural rearrangement of a nanoscale composite-structured Li_1.2_Ni_0.2_Mn_0.6_O_2_ during charge–discharge cycles

**DOI:** 10.1038/s41598-020-66411-0

**Published:** 2020-06-22

**Authors:** Keiji Shimoda, Koji Yazawa, Toshiyuki Matsunaga, Miwa Murakami, Keisuke Yamanaka, Toshiaki Ohta, Eiichiro Matsubara, Zempachi Ogumi, Takeshi Abe

**Affiliations:** 1 Office of Society-Academia Collaboration for Innovation, Kyoto University, Uji, Kyoto 611-0011 Japan; 2 JEOL RESONANCE Inc., Akishima, Tokyo 196-8558 Japan; 3 SR Center, Ritsumeikan University, Kusatsu, Shiga 525-8577 Japan; 4 Department of Materials Science and Engineering, Kyoto University, Kyoto, 606-8501 Japan; 5 Gradual School of Global Environmental Studies, Kyoto University, Katsura, Nishikyo, Kyoto 615-8510 Japan

**Keywords:** Batteries, Energy

## Abstract

Lithium- and manganese-rich layered oxides (LMRs) are promising positive electrode materials for next-generation rechargeable lithium-ion batteries. Herein, the structural evolution of Li_1.2_Ni_0.2_Mn_0.6_O_2_ during the initial charge–discharge cycle was examined using synchrotron-radiation X-ray diffraction, X-ray absorption spectroscopy, and nuclear magnetic resonance spectroscopy to elucidate the unique delithiation behavior. The pristine material contained a composite layered structure composed of Ni-free and Ni-doped Li_2_MnO_3_ and LiMO_2_ (M = Ni, Mn) nanoscale domains, and Li ions were sequentially and inhomogeneously extracted from the composite structure. Delithiation from the LiMO_2_ domain was observed in the potential slope region associated with the Ni^2+^/Ni^4+^ redox couple. Li ions were then extracted from the Li_2_MnO_3_ domain during the potential plateau and remained mostly in the Ni-doped Li_2_MnO_3_ domain at 4.8 V. In addition, structural transformation into a spinel-like phase was partly observed, which is associated with oxygen loss and cation migration within the Li_2_MnO_3_ domain. During Li intercalation, cation remigration and mixing resulted in a domainless layered structure with a chemical composition similar to that of LiNi_0.25_Mn_0.75_O_2_. After the structural activation, the Li ions were reversibly extracted from the newly formed domainless structure.

## Introduction

The world is moving towards electrification as CO_2_ emission standards have resulted in a growing battery market. Rechargeable lithium ion batteries (LIBs) have been widely used as a power source for portable devices and currently the global market for electric vehicles (EVs) demands higher power, higher energy density, longer life, and lower cost batteries. A significant amount of effort has been dedicated to the development of improved battery materials, with recent studies investigating lithium- and manganese-rich layered oxides (LMRs), which are preferred candidate materials for the next-generation LIB positive electrodes due to their high reversible capacities of ≥200 mA h g^–1^ at 2.0–4.8 V^1–10^. In contrast, several drawbacks have been reported including capacity fading and voltage decay during long-term charge–discharge cycles^6–14^. The structural factors resulting in these problems must be solved to develop stable and high power batteries.

LMRs exhibit a layered rock-salt structure commonly represented as Li[Li_(1–2*×*)/3_M_*x*_Mn_(2–*x*)/3_]O_2_ (M = Ni, Co, etc.) in single-phase notation or as *x*Li_2_MnO_3_·(1–*x*)LiMO_2_ in composite notation. Based on single-phase notation, the crystal structure can be indexed with a space group of *C*2/*m*, where the structure is homogeneous and Li ions occupy the Li layer and part of the transition-metal (TM) layer with intralayer ordering between the Li and TM ions. In contrast, the composite notation can be expressed as a mixture of *C*2/*m* and *R*–3 *m* structures, where Li_2_MnO_3_ (alternatively expressed as Li[Li_1/3_Mn_2/3_]O_2_) and LiMO_2_ exhibit common *d* spacing. These two phases are dispersed as nanoscale domains over the entire structure^15^. Unfortunately, X-ray diffraction (XRD) studies provided no clear indication of which structural model appropriately represents the real structure. Many atomic column observations using advanced scanning transmission electron microscopy (STEM) have provided evidence of two structural domains in the composite structure^16–19^, while other reports showed a homogeneous atomic column, suggesting a single-phase structure^20–22^. Solid-state nuclear magnetic resonance (NMR) spectroscopy is sensitive to cation substitution in the first and second cation coordination shells, allowing the local structure to be examined at a length scale of <5 Å in diameter. Grey *et al*. reported the nonrandom cation distribution around Li ions in Li[Li_(1–2*×*)/3_M_*x*_Mn_(2–*x*)/3_]O_2_ via ^6^Li magic-angle spinning (MAS) NMR analyses, implying a composite nature instead of homogeneous solid solution^23–25^.

Lithium deintercalation causes continuous changes of the *a* and *c* lattice parameters of LMRs^26–29^. These changes are closely associated with the charge compensation mechanism, which is reflected in the charge–discharge profile. The initial charge process exhibits a characteristic voltage profile, with a potential slope of up to ~4.5 V and subsequent irreversible plateau at ~4.5 V^1,26^. Based on X-ray absorption spectroscopy (XAS) studies, charge compensation can be achieved by the TM redox couple (e.g., the Ni^2+^/Ni^4+^ couple) during the voltage slope^19,30–32^, corresponding to the shrinkage and expansion of the *a* and *c* parameters, respectively^26–29^. In contrast, it is generally accepted that charge compensation can be achieved by oxygen (O^2−^/O^−^ couple and/or O_2_ release) during the potential plateau, where the lattice parameters remain almost unchanged^26–29,32–37^. Oxygen removal is consistent with an irreversible capacity loss observed during the initial charge–discharge cycle.

In terms of delithiation behavior of the single-phase structure model, Li ions should be uniformly extracted from the entire structure because the Li environment is homogeneous. In contrast, in the composite structure model, it would be expected that the Li ions are first extracted from the Ni^2+^-bearing LiMO_2_ domain at the potential slope, and subsequently from the Li_2_MnO_3_ domain at the plateau region^2,3^. However, previous studies have shown that Li ions are deintercalated from the Li_2_MnO_3_-like environment at potential slopes of <4.5 V^24,38^. Recently, well-considered NMR shift assignments were reported for Li_1.2_Ni_0.18_Mn_0.61_Mg_0.01_O_2_, where the local cationic configurations were compatible with the characteristic ^7^Li MAS NMR signals while satisfying local electroneutrality constraints^39^. The structural evolution based on the changes in NMR signals during the first electrochemical cycle was also discussed, but the mechanism remains a source of debate due to the complicated spectral deconvolution including spinning sideband (SSB) manifolds in that study. Magic-angle turning phase-adjusted sideband separation (MATPASS) is a recently developed NMR technique and its sheared projection, pj-MATPASS spectrum, can provide well-resolved isotropic signals without the SSB manifolds^40^. Subsequent studies have reported the delithiation/lithiation dynamics of different Li sites in various LMR cathodes (Li_2_MnO_3_, Li_1.2_Ni_0.2_Mn_0.6_O_2_, and Li_1.2_Ni_0.13_Co_0.13_Mn_0.54_O_2_) using operando NMR and pj-MATPASS NMR techniques. These studies suggested that the Li ions in the TM layer were preferentially extracted during the first 20% of the charging and Li ions in both the Li and TM layers were subsequently removed at similar rates^41^. However, the detailed spectral evolutions of each signal component in the pj-MATPASS spectra were not discussed. Herein, synchrotron-radiation XRD, soft XAS, and pj-MATPASS NMR techniques were used to examine the delithiation behavior of a composite-structured Li_1.2_Ni_0.2_Mn_0.6_O_2_ in a semi-quantitative manner, and the sequential delithiation from the composite domains during the initial charge was demonstrated. The structural evolution after the initial charge–discharge cycle was also briefly discussed.

## Results and discussion

### Structural evolution of Li_1.2_Ni_0.2_Mn_0.6_O_2_ during the 1^st^ charge–discharge cycle

**Figure 1:**
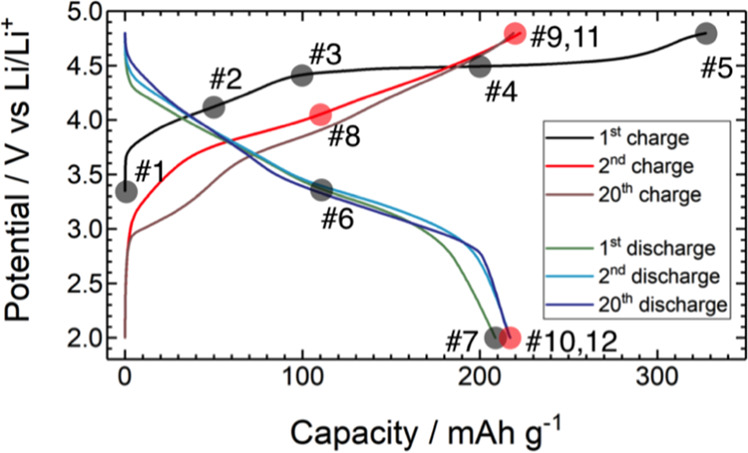
Charge–discharge profiles of the Li//Li_1.2_Ni_0.2_Mn_0.6_O_2_ cell at the 1^st^, 2^nd^, and 20^th^ cycles. The electrochemical measurements were performed at 50 °C between 2.0 and 4.8 V vs. Li/Li^+^ at a constant current of 20.5 mA g^−1^. The sampling points are marked.

**Figure 2:**
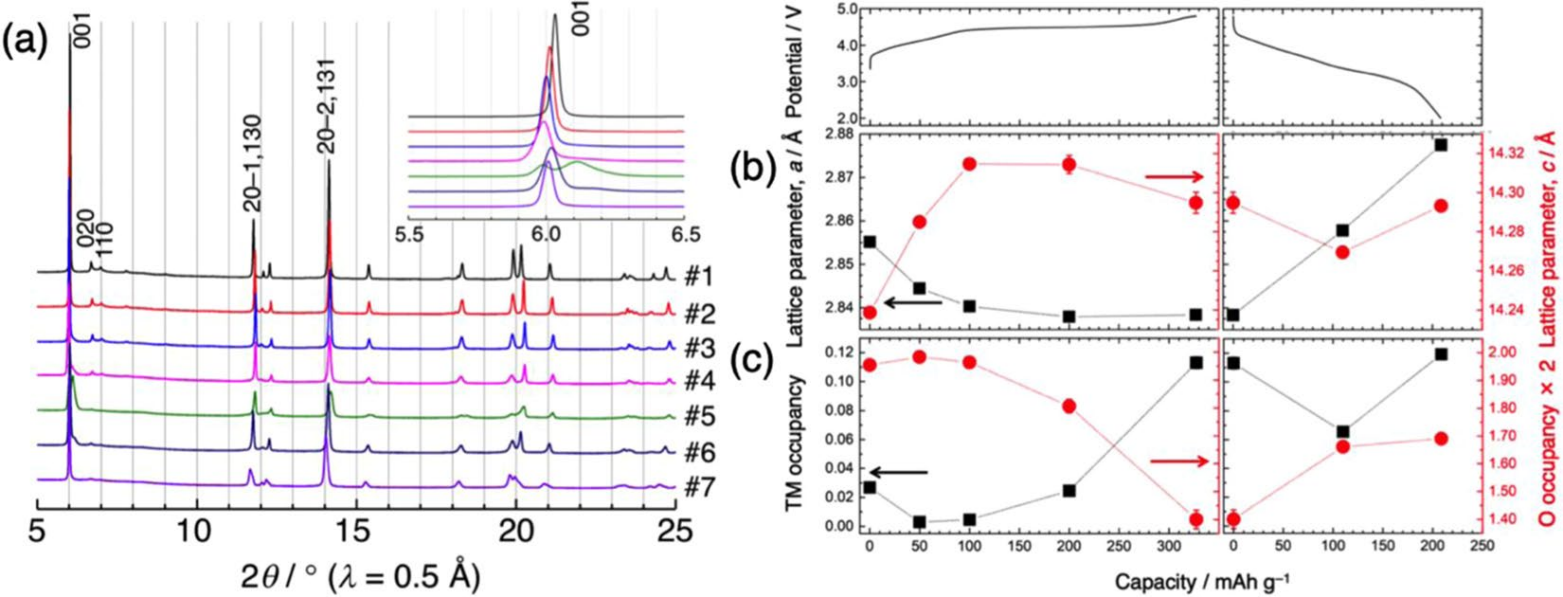
(**a**) SR-XRD profiles of the Li_1.2_Ni_0.2_Mn_0.6_O_2_ electrode at the 1^st^ cycle. The strongest 001 diffraction peak was indexed with *C*2/*m* and is enlarged in the inset. The sample numbers are described in Fig. 1. (**b**) Evolution of the *a* and *c* lattice parameters and (**c**) TM and O occupancies during the 1^st^ cycle. Error bars in (**b,c**) indicate the standard uncertainties (±3σ) derived from the Rietveld refinements (smaller than the symbols in most cases).

Figure 1 shows the charge–discharge profiles of Li_1.2_Ni_0.2_Mn_0.6_O_2_ for the 1^st^, 2^nd^, and 20^th^ cycles (complete profiles and corresponding d*Q*/d*V* curves are provided in Fig. S1). Sampling points for the structural analyses are clearly marked. Figure 2(a) shows the SR-XRD profiles for the 1^st^ charge–discharge cycle. Based on single-phase notation, the pristine material was indexed with a space group of *C*2/*m* and the lattice parameters were refined as *a* = 4.951 Å, *b* = 8.558 Å, *c* = 5.028 Å, and *β* = 109.213° (#1)^42,43^, although the high-angle annular dark field (HAADF)-STEM image of the material showed a composite structure, where the atomic columns of the TM layer of Li_2_MO_3_ and LiMO_2_ are clearly discernible (Fig. S2(a))^42^. Close inspection of the strongest 001 diffraction peak provides clear suggestions regarding average crystal structure modification during the delithiation/lithiation process (Fig. 2(a), inset). The 001 peak moves to a lower 2*θ* value during charging to 100 mA h g^−1^ (potential slope region, #2 and 3). At 200 mA h g^–1^, at a midway point of the potential plateau region, the peak width broadens with a position close to that at 100 mA h g^–1^ (#4). Subsequently, the peak splits into two separate peaks at 4.8 V (#5), which then merge into a single peak during the lithiation, indicating that the structural modification is reversible (#6 and 7). A similar type of peak splitting at a high potential has been reported by Koga *et al*., who attributed the two peaks at lower and higher 2*θ* to bulk and degraded surface phases, respectively^44^. Herein, the peak at higher 2*θ* is close to the strongest 111 diffraction peak of the Li-poor cubic spinel Li_0.05_Mn_2_O_4_ (*λ*-MnO_2_, *a* = 8.0445 Å)^45,46^. The formation of a spinel structure upon charging was substantiated via electron microscopy techniques^16,47,48^. The superlattice peaks characteristic of *C*2/*m* were observed for the pristine material in the 2*θ* range of 6.3°–11.0°. The intensity of these peaks gradually decreased during the delithiation process, which was mostly irreversible (Fig. S3). This indicates that the Li/TM honeycomb ordering in the TM layer was permanently lost by Li extraction from the original structure.

Rietveld refinements with the space group of *R*–3 *m* (*Fd*–3 *m* for the second phase at 4.8 V) were performed for simplicity, although the refinements with the *C*2/*m* structure may yield better Reliability factors (*R*_p_ and *R*_wp_). For the *R*–3 *m* structure, the oxygen occupancy at the 6*c* Wyckoff position and the TM ion occupancy in the Li and TM layers (3*a* and 3*b* positions, respectively) with fixed isotropic atomic displacement parameters were refined in addition to the *a* and *c* lattice parameters. For the *Fd*–3 *m* structure, only the *a* lattice parameter was refined and the superlattice peaks in the 2*θ* range of 6.3°–11.0° were omitted during the refinement procedures. The final *R*_p_ and *R*_wp_ values were <4.6% and <7.0%, respectively, for all samples. Figure 2(b) shows the variations of the *a* and *c* parameters during the 1^st^ cycle where *a* decreases and *c* increases over the potential slope region. The contraction of the *a*-axis and elongation of the *c*-axis are a consequence of Ni oxidation, forming smaller ionic radii and increasing electrostatic repulsion between the close-packed oxygen layers due to Li extraction^26–29,44^. In contrast, the variations of the lattice parameters were negligible during the potential plateau region, suggesting charge compensation by the oxygen redox mechanism^26–29,44^. The nanoscale phase separation from the composite layered structure to layered and spinel(-like) structures was observed only at 4.8 V and the mass ratio of the two phases was estimated as 66:34 from the refinement. Recently, it was reported that phase separation at high voltage is dependent on particle size^29^ where two-phase behavior was observed in samples with large particles (10 μm), but one-phase behavior was found for samples with sub-micrometer particles. Herein, the sample exhibited a ~5 μm secondary particle size, so phase separation was observed. During the lithiation process, the *a* parameter largely increases, while the *c* parameter slightly decreases. The former is associated with reduction of the TM ions, and the latter is likely due to incomplete lithium uptake. Figure 2(c) shows the TM ion occupancy variations in the Li layer for the *R*–3 *m* structure, suggesting increased TM migration into the Li layer after activation. Spectroscopic evidence of the migration was also provided by resonant X-ray diffraction spectroscopy (RXDS)^49^. The variation of oxygen occupancy is also shown in Fig. 2(c). Oxygen atoms exhibit relatively weak X-ray scattering power compared to TM atoms, but the high resolution and high flux X-ray beam provided a general trend for oxygen occupancy in the refinement results. The oxygen occupancy remained nearly constant during the potential slope region, but decreased in the plateau region, suggesting oxygen loss from the layered structure. The oxygen loss and TM migration was correlated as the oxygen vacancy may facilitate TM migration^50^. These results indicate that the layered structure retained at 4.8 V is highly defective, which may transform into a spinel-like structure with further delithiation. The oxygen occupancy is partially recovered during lithiation. A similar behavior was previously reported, and it is considered that the oxygen vacancies are partly refilled by extracting the oxygen from surface film components (for example, Li_2_CO_3_) on the particle, which are caused by electrolyte decomposition^51^. Alternatively, the peroxide moiety formed in the charged material may account for the reversibility of oxygen occupancy^51^. Such O-O dimer species locate at the interstitial positions, which are not explicitly taken into account for structural refinements. Li reinsertion breaks the dimer bond, and the oxygen atoms may go back to the 6*c* site in the discharged material. A possibility of the formation of peroxide species is discussed in the next section.

**Figure 3:**
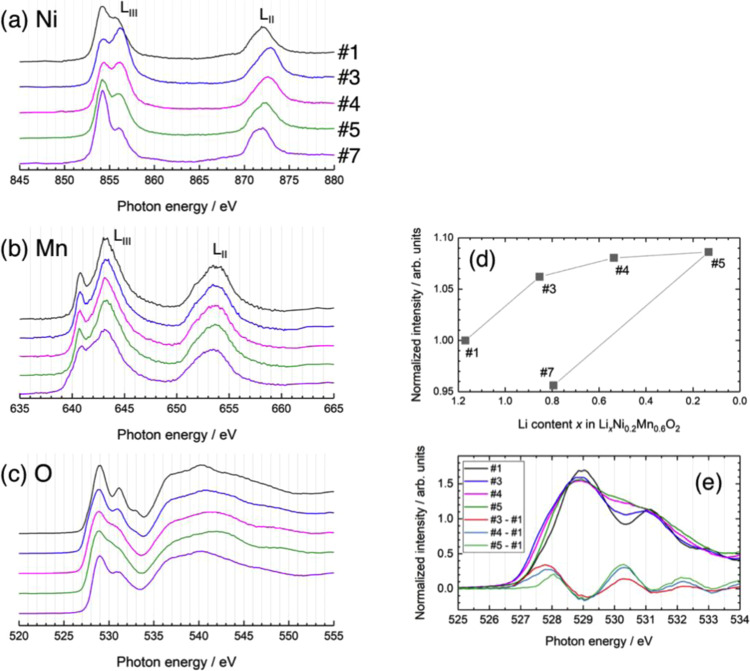
(**a**) Ni L-, (**b**) Mn L-, and (**c**) O K-edge XAS spectra of the Li_1.2_Ni_0.2_Mn_0.6_O_2_ electrode at the 1^st^ cycle. (**d**) Evolution of the integrated intensity in the pre-edge region of the O K-edge XAS spectra. (**e**) Difference spectra of the pre-edge region of the O K-edge XAS spectra.

Figure 3 shows the Ni, Mn L_II,III_-, and O K-edge XAS spectra acquired in partial fluorescence yield (PFY) or inverse partial fluorescence yield (IPFY) mode for the 1^st^ cycle. The TM L-edge XAS directly probes the electron dipole transition from the 2p core level to 3d unoccupied states and is sensitive to the valence and coordination states. The L-edge spectra split into L_III_ and L_II_ lines due to the spin-orbit interaction of 2p_3/2_ and 2p_1/2_ core electrons. The Ni L_III_-edge spectrum of the pristine sample (#1) was similar in shape to those of NiO and LiNiO_2_^32,52,53^. The average Ni valence was determined to be 2.3+ from the Ni K-edge XAS spectrum^49^. At the end of the potential slope region, the lower energy peak of the L_III_-edge decreased in intensity while the higher energy peak increased, with the L_II_-edge peak shifting to a higher energy, indicating an increase in Ni oxidation state (#3). However, the higher energy peak of the L_III_-edge decreased in relative intensity, while the L_II_-edge peak shifted back to a lower energy at the potential plateau, suggesting partial reduction of Ni (#4 and 5). The L_III_-edge spectrum of the sample discharged to 2.0 V (#7) was almost identical to that of NiO, indicating a valence state close to 2+. From the spectral analysis by the linear combination fitting, in which it was assumed that spectra #3 and #7 are representative of Ni^4+^ and Ni^2+^ states, respectively, the Ni valence state was estimated to be 3+ at 4.8 V (Table S1). The decreased Ni valence state at high voltages has been previously reported by several researchers^32,54^. Hy *et al*. suggested that this reduction behavior was associated with the preferential hybridization of Ni with activated oxygen species formed at high potentials^54^. The Mn L_III_-edge spectrum of the pristine material resembled that of Li_2_MnO_3_^54–56^, indicative of the tetravalent state, as expected (Fig. 3(b)). The oxidation state of Mn remained almost constant during charging, in contrast to previous studies of Li_2_MnO_3_, where the valence state was reduced to 3.5+ at 4.8 V^55,56^. This also departs from the decreased Ni valence mentioned above. Ni has a larger electronegativity and is expected to strongly hybridize with the activated oxygen species preferentially over the Mn ion^54^. In contrast, Mn valence decreased to 3.5+ at 2.0 V, based on the spectral analysis using reference spectra of MnO (Mn^2+^), Mn_2_O_3_ (Mn^3+^), and Li_2_MnO_3_ (Mn^4+^) (Table S1). The O K-edge spectra showed pre-edge peaks between 527 and 534 eV and a white line above 534 eV, which come from the O 2p unoccupied states hybridized with TM 3d and 4 s,p states, respectively (Fig. 3(c)). Detailed examination indicated that the spectral intensity of the pre-edge area changes in two steps during charging and its intensity evolution between 525 and 534 eV is plotted in Fig. 3(d). The intensity increased during the potential slope but remained nearly constant over the plateau region. The difference spectra between spectra #1, #3, and #5 are provided in Fig. 3(e). An intensity increase at the lower energy side (~528 eV) of the 529 eV peak was observed until the end of the potential slope^36^. This was also observed in the O K-edge spectrum for Li_1–*x*_MO_2_ and was attributed to the increased contribution of the ligand hole in the LiMO_2_ domain^54,57–61^. This spectral change is associated with a valence increase of Ni ions hybridized with O ions, suggesting a cation-anion dual charge compensation process^43^. The 528 eV component decreased at 4.8 V, and a new peak at ~530 eV increased in intensity. The former behavior is due to the valence decrease of Ni, as shown in the Ni L_III_-edge spectra, while the latter is unrelated to the valence change of the TM ions in the LiMO_2_ domain and is instead related to Li_2_MnO_3_ domain structural changes. Previous studies reported a similar intensity increase at 530 eV for delithiated Li_2_MnO_3_^55,56^. This is consistent with the assertion that Li ions are extracted from the Li_2_MnO_3_ domain over the potential plateau^2,3^. The intensity increase at 530 eV likely corresponds to the formation of peroxide-like oxygen in the Li_2_MnO_3_ domain, because the peak position is similar to that of Li_2_O_2_^33,55,62^. Moreover, a recent study examined the O-O vibrational mode typical of the peroxide moiety in the charged Li_1.2_Ni_0.2_Mn_0.6_O_2_ using *in situ* surface-enhanced Raman scattering (SERS)^63^. Upon discharge, the spectral features in the pre-edge region were similar to those of pristine material, indicating that the electronic states of O ions were mostly recovered.

**Figure 4:**
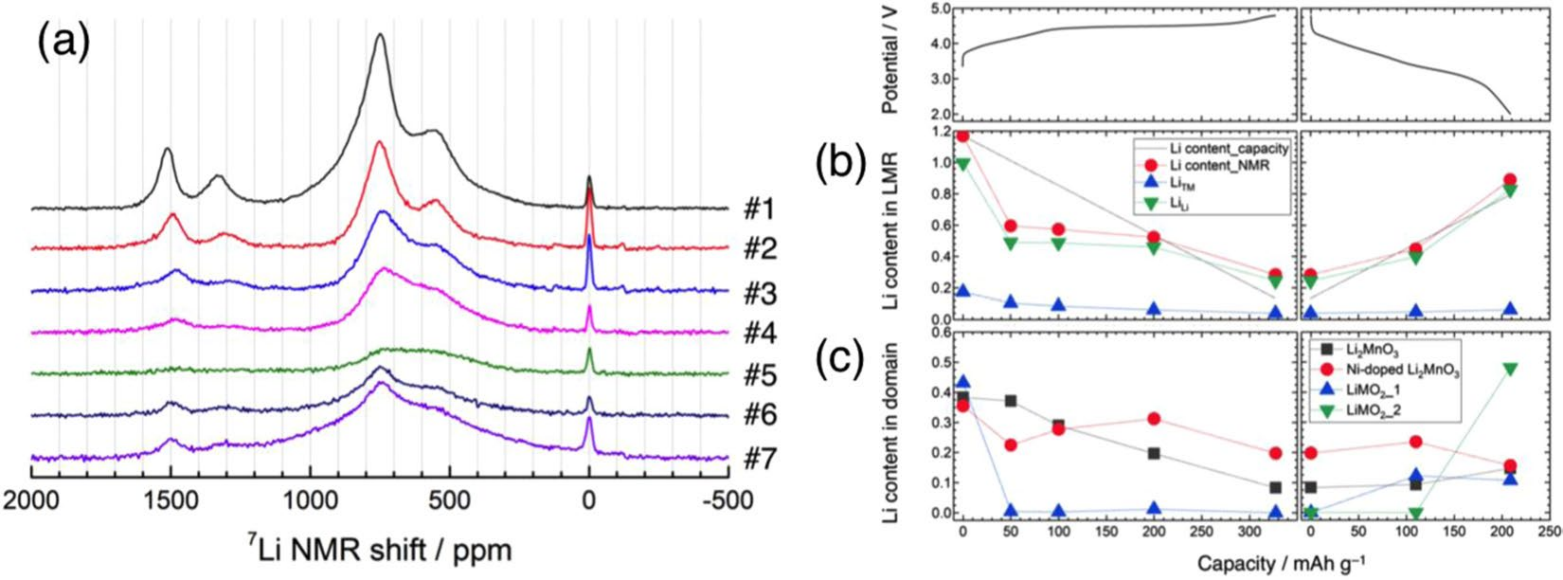
(**a**) ^7^Li pj-MATPASS NMR spectra of the Li_1.2_Ni_0.2_Mn_0.6_O_2_ electrode at the 1^st^ cycle. (**b**) Evolution of the total Li content derived from the ^7^Li NMR intensity (Li_TM_ and Li_Li_) and electrochemical measurements. (**c**) Evolution of Li contents in the various domains (Li_2_MnO_3_-, Ni-doped Li_2_MnO_3_-, and LiMO_2_-like domains) during the 1^st^ cycle.

Figure 4(a) shows the pj-MATPASS NMR spectra of Li_1.2_Ni_0.2_Mn_0.6_O_2_ electrodes during the 1^st^ cycle. A comparison of NMR spectra of the pristine material, acquired using different techniques and under different experimental conditions, shows that only the pj-MATPASS spectrum provides isotropic signals without SSB manifolds even at a moderate sample spinning rate of 30 kHz and high magnetic field of 14.1 T (Fig. S4). The ^7^Li signals for Li_1.2_Ni_0.2_Mn_0.6_O_2_ were split into 6 pseudo-Voigt components (Fig. S5). There are different local environments, and some components are correlated with each other, suggesting that these local environments are attributable to different phases (domains) in Li_1.2_Ni_0.2_Mn_0.6_O_2_. Based on previous studies, where the additive nature of the Fermi contact shift and local cationic configurations (bond angles of Li-O-TM) were summed^39,64^, each component was assigned. The predominant peak components at 749 and 1514 ppm were exclusively assigned to Li ions in the Li and TM layers of the Ni-free Li_2_MnO_3_-like region in Li_1.2_Ni_0.2_Mn_0.6_O_2_, respectively. These components were close to those of Li_2_MnO_3_^41,56^, and additional signals appeared with increasing Ni contents in Li[Li_(1–2__*x*__)/3_Ni_*x*_Mn_(2–__*x*__)/3_] O_2_ (Fig. S6). The peak components at 553 and 1331 ppm were attributed to Li ions in the Li and TM layers of the Ni-doped Li_2_MnO_3_-like region, respectively^24,25,64^. The latter Li ions are surrounded by 6 TM ions in Ni:Mn = 1:5 in the TM layer. Because Ni ions in an octahedral environment exhibit lower spins (*S* = 1, 1/2, and 0 for Ni^2+^, Ni^3+^, and Ni^4+^, respectively) compared to *S* = 3/2 for Mn^4+^, yielding weaker hyperfine interactions, the ^7^Li signals in the Ni-doped Li_2_MnO_3_ (Li_2_[Ni_1/6_Mn_5/6_]O_3_-like) domain appeared at lower frequencies (553 and 1331 ppm) compared to those in the Ni-free domain (749 and 1514 ppm)^64^. The 791 ppm component was tentatively assigned to the ^7^Li signal from the Ni-containing LiMO_2_ domain due to the lack of the corresponding ^7^Li signal from the TM layer. Some assignments differed from those in a recent study^39^, where the peaks at 576 and 946 ppm (corresponding to the peaks at 553 and 791 ppm observed herein) were assigned oppositely. The remaining 352 ppm component was not observed in previous studies^39,64^, and was tentatively ascribed to Li ions in the Li layer of the Li_2_[Ni_2/6_Mn_4/6_]O_3_-like domain. This contribution is negligible and is merged with the discussion of the 553 ppm peak below. Finally, the sharp signal at 0 ppm arises from impurity phases such as Li_2_CO_3_ and organic lithium salts.

The HAADF-STEM images visualized that the atomic columns of the TM layer characteristic of Li_2_MO_3_ and LiMO_2_-like structure were alternately stacked over several nm (Fig. S2(a))^42^. This indicates that Li_1.2_Ni_0.2_Mn_0.6_O_2_ consists of composite domains adopting Li_2_MO_3_- and LiMO_2_-based layered structures. The ^7^Li pj-MATPASS spectra provided detailed information about chemical composition of the composite domains in Li_1.2_Ni_0.2_Mn_0.6_O_2_. The ratio of Li ions in the Li and TM layers, Li_TM_/Li_Li_, was estimated from the ^7^Li pj-MATPASS spectrum for the pristine material (0.17). Therefore, the bulk chemical composition of the pristine material was re-expressed as Li[Li_0.17_Ni_0.21_Mn_0.59_]O_2_ (almost identical to that of Li_1.2_Ni_0.2_Mn_0.6_O_2_) including the Ni and Mn valence states estimated from previous XAS results (2.3+ and 4+, respectively)^49^. The composition and ratio of the Ni-free and Ni-doped Li_2_MnO_3_ (Li_1.33_Mn_0.67_O_2_) and LiMO_2_ domains were estimated as 0.28Li_1.37_Mn_0.63_O_2(–*δ*)_ (similar to Li_1.33_Mn_0.67_O_2_), 0.28Li_1.26_Ni_0.11_Mn_0.57_O_2_, and 0.43LiNi_0.41_Mn_0.57_O_2_, considering the experimental ^7^Li signal ratio Li_TM_/Li_Li_ of each domain (0.37, 0.26, and 0, respectively), and cation vacancies as well as the assumption that the Ni ions are tetravalent in the Ni-doped Li_2_MnO_3_-like domain with Ni:Mn = 1:5 (see also Supplementary Information). The estimated phase ratio is comparable to the composite notation of Li_1.2_Ni_0.2_Mn_0.6_O_2_, 0.6Li_1.33_Mn_0.67_O_2_·0.4LiNi_0.5_Mn_0.5_O_2_. It should be emphasized that the Ni-doped Li_2_MnO_3_-like domain, Li_1.26_Ni_0.11_Mn_0.57_O_2_, is a dominant component in the material, acting as a boundary buffer phase between the Li_2_MnO_3_ and LiMO_2_ domains^39^. The composition and ratio of these domains differed from those estimated in ref. ^39^ with a similar bulk composition, Li_1.2_Ni_0.18_Mn_0.61_Mg_0.01_O_2_, mostly due to different peak assignments. It should be noted that the composite structure of differently composed domains in Li_1.2_Ni_0.2_Mn_0.6_O_2_ may introduce chemical inhomogeneity within a nanoscale particle. The inhomogeneous Ni distribution relative to Mn and O is highlighted in the energy-dispersive X-ray spectrometry (EDS) mapping images of a single particle (Fig. S2(b)). The LiNi_0.41_Mn_0.57_O_2_ domain may be concentrated in the Ni-rich region.

The ^7^Li pj-MATPASS and its difference spectra indicate that Li extraction from each signal component is not constant (Figs. S5 and S7). Figure 4(b) shows the integrated intensity variation of ^7^Li signals of Li_1.2_Ni_0.2_Mn_0.6_O_2_ for the 1^st^ cycle (see Fig. S8 for each component). Herein, the NMR-based composition, Li[Li_0.17_Ni_0.21_Mn_0.59_]O_2_ (theoretical capacity: 369.5 mA h g^–1^), was used instead of the ICP-AES-based composition, Li_1.25_Ni_0.20_Mn_0.55_O_2–*δ*_, or the ideal composition, Li_1.2_Ni_0.2_Mn_0.6_O_2_, for consistency. Total ^7^Li signals decreased in intensity during charging. It should be noted that the Li contents at 50 and 100 mA h g^–1^ were significantly underestimated compared to those calculated from the delivered current densities and similar behaviors were reported for LiCoO_2_ and LiNi_0.5_Mn_1.5_O_4_^65,66^. This abrupt intensity reduction during the early stages of delithiation arises from the localized nature of the electrons on paramagnetic ions and nearby Li^+^ ions, causing rapid relaxation of all nearby ^7^Li nuclear spins before signal acquisition. In contrast, the Li content in the sample at 4.8 V was slightly overestimated, indicating that the delivered charging capacity includes some contribution from side reactions such as electrolyte decomposition at high potentials. During discharging, the Li contents increased almost linearly. Figure 4(b) shows that the Li ions were extracted from the Li and TM layers of Li[Li_0.17_Ni_0.21_Mn_0.59_]O_2_. The Li_TM_/Li_Li_ ratio remained almost constant during charging, but decreased gradually during discharging (Fig. S9). This suggests that the Li ions cannot easily be reinserted into the TM layer of the discharged material.

The Li content in each domain during the 1^st^ charge–discharge cycle is plotted in Fig. 4(c). The LiMO_2_ domain content is drastically reduced at the 50 mA h g^–1^ charge, leading to the abrupt intensity reduction discussed above (Fig. 4(b)). Therefore, it is clear that Li ions are preferably extracted from the LiMO_2_ domain over the potential slope region. From the XAS results (Fig. 3(a) and ref. ^49^), Li extraction is electronically compensated by the valence increase of Ni in the LiMO_2_ domain. It should be noted that some ^7^Li signals were lost in the Ni-free and Ni-doped Li_2_MnO_3_ domains over the potential slope region (Figs. 4(a) and S7). This signal loss is likely fictitious, resulting from the strong interaction with the localized paramagnetic spins in the nearby LiMO_2_ domain. Li ions are subsequently deintercalated from the Li_2_MnO_3_ domain during the potential plateau with charge compensation via oxidation of lattice oxygen and O_2_ release, as described above. At 4.8 V, a broad ^7^Li signal remained at ~600 ppm (Fig. 4(a), #5), suggesting that Li ions are mainly retained in the disordered Li layer of the Ni-doped Li_2_MnO_3_ domain. It should be noted that some researchers have attributed the broad signal at 500–600 ppm to Li ions at tetrahedral sites^38,64^. Considering the phase separation observed in the XRD profile at 4.8 V (Fig. 2(a)), delithiation from the Li_2_MnO_3_ domain involves the oxygen loss and the formation of a spinel-like phase^56^, and the remaining Li ions are most likely retained in the layered structure consisting of the Ni-doped Li_*x*_MnO_3_ domain. The composition of the material at 4.8 V was determined to be Li_0.24_[Li_0.04_Ni_0.21_Mn_0.59_]O_2–*δ*_. During discharging, Li ions are mainly reinserted into the LiMO_2_ domain and a new broad component appears when discharged to 2.0 V (Fig. S7). This was ascribed to a ^7^Li signal from a newly formed LiMO_2_ phase, where the Ni and Mn ions in the TM layer are randomly distributed in various configurations. This new phase becomes dominant and the spectrum of the discharged material at 2.0 V differed significantly from that of the pristine material. The chemical composition at 2.0 V can be expressed as Li_0.83_[Li_0.06_Ni_0.21_Mn_0.59_]O_2–*δ*’_, while the ideal composition would be Li_~1.0_Ni_0.25_Mn_0.75_O_2_ (expected reaction: Li_1.2_Ni_0.2_Mn_0.6_O_2_ → 0.8LiNi_0.25_Mn_0.75_O_2_ + 0.4Li + 0.2O_2_↑, where the oxygen loss was evidenced in Fig. 2(c)). These results indicate that delithiation proceeds sequentially from the LiMO_2_ and Li_2_MO_3_ domains in the Li_1.2_Ni_0.2_Mn_0.6_O_2_ composite structure. This non-uniform Li extraction from the composite material was demonstrated for Li_1.2_Mn_0.4_Fe_0.4_O_2_ by elemental mapping analyses of electron energy loss spectroscopy (EELS)-STEM images^67^. The structure of Li_1.2_Ni_0.2_Mn_0.6_O_2_ after the initial cycle changed to a domainless single phase LiMO_2_, although some Li_2_MnO_3_ domain remained, indicating that irreversible structural changes occur during the initial charge–discharge process.

The ^7^Li signal at 1514 ppm is relatively sharp and well separated from other components, allowing the peak position changes to be confirmed with high confidence (Fig. S10). Over the potential slope region, the peak position shifted towards lower frequencies corresponding to the Ni valence increase. The valence state of Ni in the LiMO_2_ domain changed from Ni^2+^ (*S* = 1) to Ni^4+^ (*S* = 0), reducing the Fermi contact interaction and shifting the peak position to lower frequencies. In contrast, no peak shift was observed over the potential plateau region, indicating that the TM ions were not involved in charge compensation. During the discharging process, the peak position returns, to some extent, to higher frequencies due to TM ion reduction. These changes are qualitatively consistent with that of the *a* lattice parameter.

Therefore, it can be concluded that the irreversible potential plateau in the initial charge–discharge cycle corresponds to a structural change (i.e., structural activation) from a composite structure of Li_2_MO_3_ and LiMO_2_ domains to a single-phase (domainless) structure. This is associated with cation mixing (i.e., migration of TM ions) during the formation of a spinel-like structure at a high potential. Upon back-transformation to a layered structure during discharging, the TM ions move back to positions different from their original positions in the TM layer, and the TM ions are uniformly dispersed, resulting in a domainless structure.

### Structural evolution after the initial charge–discharge cycle

The structure of Li_1.2_Ni_0.2_Mn_0.6_O_2_ after the initial charge–discharge cycle will be briefly discussed. The charge–discharge profiles for the 2^nd^ and 20^th^ cycles are shown in Fig. 1. The capacity fade was small but gradual voltage fading was observed up to the 20^th^ cycle (Fig. S1). The charging and discharging capacities at the 20^th^ cycle were 218.8 and 216.8 mA h g^–1^, respectively.

**Figure 5:**
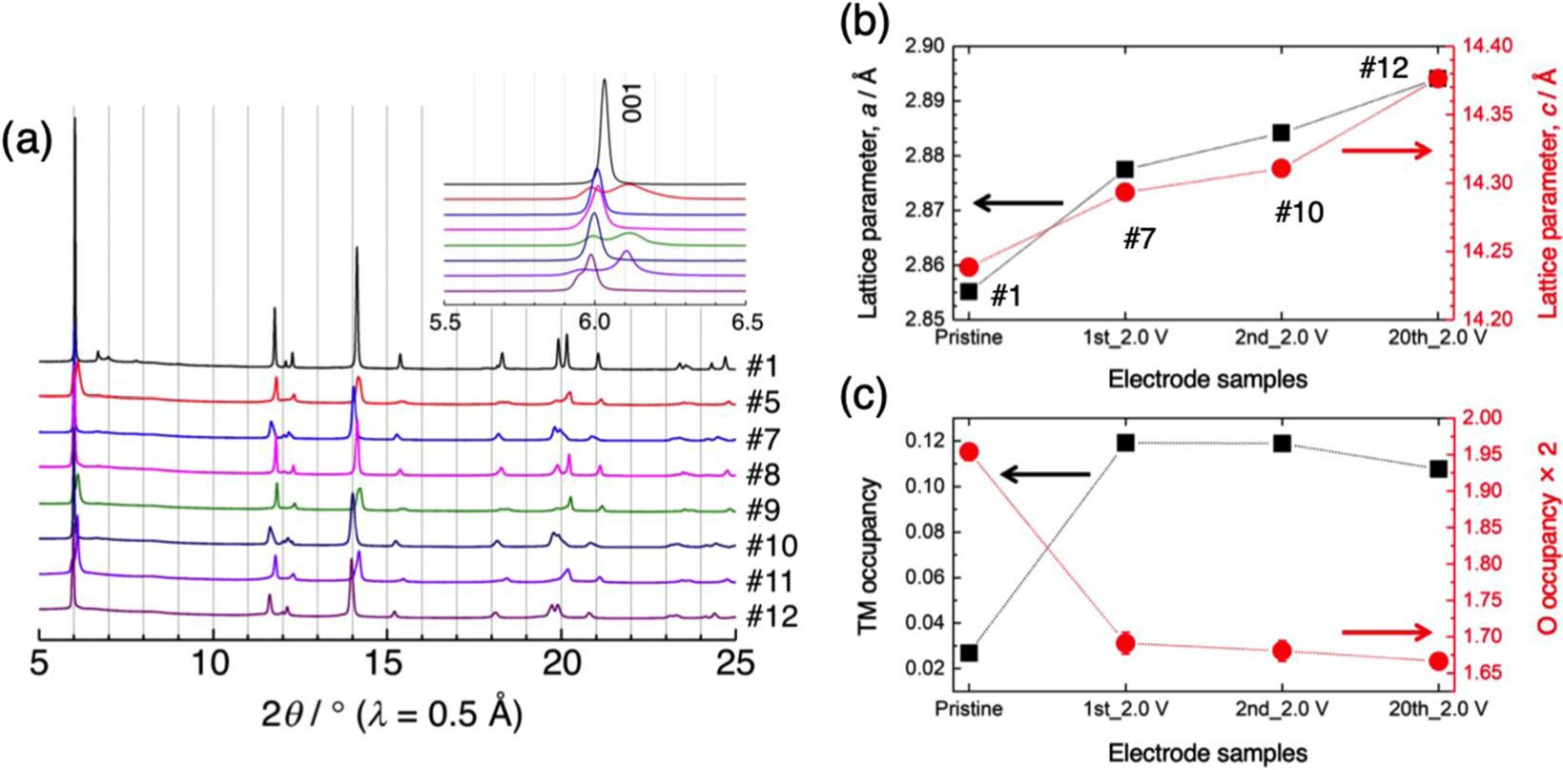
(**a**) SR-XRD profiles of the Li_1.2_Ni_0.2_Mn_0.6_O_2_ electrode at the 1^st^, 2^nd^, and 20^th^ cycles. The strongest 001 diffraction peak is enlarged in the inset. (**b**) Evolution of the *a* and *c* lattice parameters and (**c**) TM and O occupancies for the pristine, 1^st^, 2^nd^, and 20^th^ discharged samples. Error bars in (**b**,**c**) indicate the standard uncertainties (±3σ) derived from the Rietveld refinements (smaller than the symbols in most cases).

The SR-XRD profiles of the fully charged and discharged samples at the 1^st^, 2^nd^, and 20^th^ cycles are shown in Fig. 5(a). The diffraction profiles of the 2^nd^ charged and discharged samples (#9 and 10) are quite similar to those of the 1^st^ cycle (#5 and 7). Even at the 20^th^ charge–discharge cycle, the 001 diffraction peak of the charged sample was split (see Fig. 5(a), inset), merging into a single peak for the discharged sample (#11 and 12). We note that each peak at lower and higher 2*θ* of the split 001 diffraction peak in the 20^th^ charged sample was broader and narrower, respectively, than the corresponding peaks in the 1^st^ and 2^nd^ charged samples. This may indicate the decrease in domain size (or crystallinity) of layered structure phase and the increase in domain size (or crystallinity) of spinel phase. In addition, a small shoulder at the lower 2*θ* side of the 001 diffraction peak in the 20^th^ discharged sample may be a remnant peak of the layered structure observed in the charged state. These results indicate that the structural changes are reversible after structural activation during the initial cycle. This structural stability is significantly different from that of Li_2_MnO_3_, which changes from a layered structure to a highly disordered spinel-like structure in 20 cycles^56^. It should be noted that the spinel-like phase was observed at 4.8 V in the 20^th^ cycle, although the Li_2_MnO_3_ domain completely disappeared, as discussed below. Therefore, this spinel-like structure results from the domainless layered structure at the high voltages^68^. Figure 5(b) shows the lattice parameters changes of the pristine and discharged samples at the 1^st^, 2^nd^, and 20^th^ cycles. The domain structure in the pristine material changes to a domainless structure after the 1^st^ discharge and both the *a*- and *c*-axes were correspondingly expanded. The lattice parameters further increased for the 2^nd^ and 20^th^ discharged materials. The extent of TM ion migration (to the Li layer) and oxygen site occupancy remained nearly constant after the 1^st^ cycle (Fig. 5(c)). Therefore, the increases in *a*- and *c*-axes are likely caused by the decreased average oxidation state of TM ions and decreased Li content, respectively.

**Figure 6:**
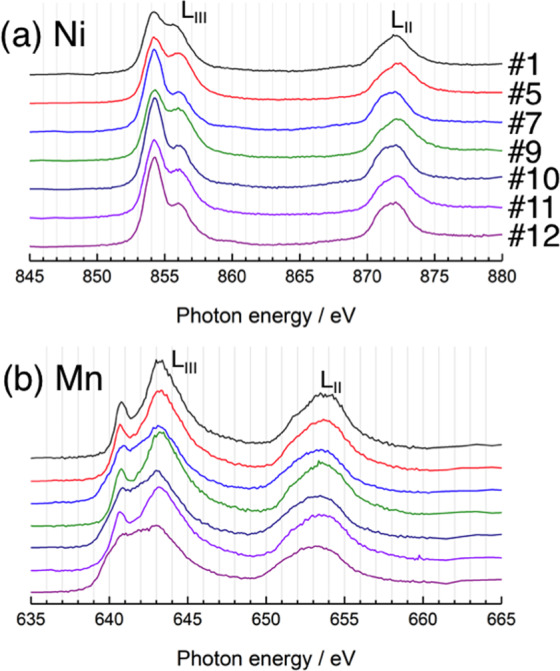
(**a**) Ni L- and (**b**) Mn L-edge XAS spectra of the Li_1.2_Ni_0.2_Mn_0.6_O_2_ electrode at the 1^st^, 2^nd^, and 20^th^ cycles.

Figure 6 shows the Ni and Mn L_II,III_-edge XAS spectra for the 1^st^, 2^nd^, and 20^th^ charged and discharged samples. The Ni valence was divalent for all discharged materials. The Ni valence was estimated to be 3.0+ and 2.6+ in the 2^nd^ and 20^th^ charged materials, respectively (Table S1). The average Mn valence in the pristine and 1^st^, 2^nd^, and 20^th^ discharged states gradually decreased from 4.0+ to 3.5+, 3.4+, and 3.3+, respectively (Table S1), which is consistent with increased *a*-axis, indicating that Mn ions are redox active after the initial cycle. This is consistent with a previous study using hard X-ray photoelectron spectroscopy (HAXPES)^43^, while these soft XAS spectra include some contributions from the active material surface degradation. The present results suggest that Ni ions become redox inactive at higher voltages (> ~3.2 V for Ni^2+^/Ni^4+^ redox couple), and Mn ions become redox active at lower voltages (<~3.2 V for Mn^3+^/Mn^4+^ redox couple) during repeated cycles. This is involved in voltage fading behavior shown in Fig. S1(b).

**Figure 7:**
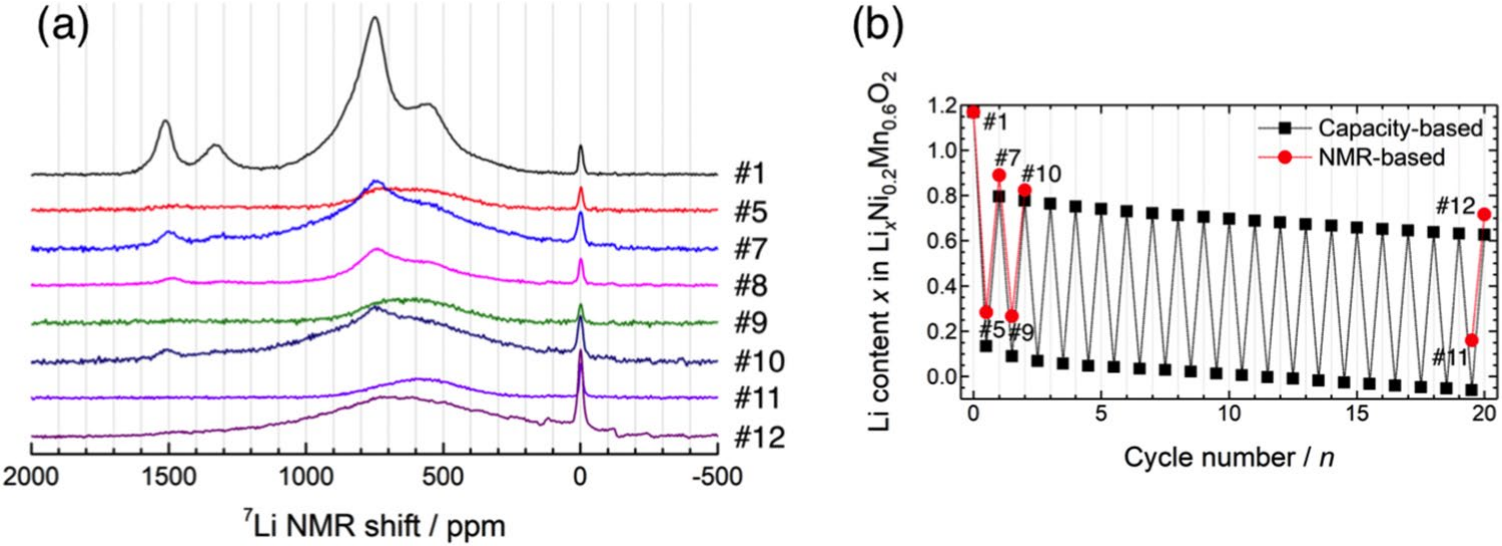
(**a**) ^7^Li pj-MATPASS NMR spectra of the Li_1.2_Ni_0.2_Mn_0.6_O_2_ electrode at the 1^st^, 2^nd^, and 20^th^ cycles. (**b**) Evolution of the total Li content derived from the ^7^Li NMR intensity and electrochemical measurements up to 20^th^ cycle.

Figure 7(a) shows the ^7^Li pj-MATPASS spectra of the 1^st^, 2^nd^, and 20^th^ charged and discharged samples, where comparison of the charged (#5, 9, and 11) and discharged samples (#7, 10, and 12) shows no significant differences in the spectra, except for consumption of the residual signals of the Li_2_MnO_3_ component at the 20^th^ cycle. Therefore, the Li ions were reversibly extracted and inserted into the domainless layered structure after the initial cycle. Broad signals centered at 585 and 630 ppm were observed in the 20^th^ charged and discharged materials, respectively. The higher frequency isotropic shift corresponds to the lower average oxidation state of TM ions, with higher spin numbers of *S* = 2 and 1 (for Mn^3+^ and Ni^2+^, respectively) compared to *S* = 3/2 and 0 (for Mn^4+^ and Ni^4+^, respectively). The Li contents estimated from the signal intensities of the 20^th^ charged and discharged samples were slightly overestimated relative to that calculated from the charging and discharging capacities (Fig. 7(b)). Li ions were observed in LiNi_0.25_Mn_0.75_O_2_ in the 20^th^ charge (#11), although the Li content calculated from the capacity at 4.8 V should be empty. These results indicate that the long-cycled charge–discharge capacity includes significant contributions from accumulative side reactions and that NMR spectroscopy is a reliable tool for directly measuring the Li content in active materials.

## Conclusions

Based on the composite structure concept of LMRs, the delithiation behavior of Li_1.2_Ni_0.2_Mn_0.6_O_2_ was reexamined during charge–discharge cycles. It was confirmed that the pristine material contains a composite layered structure composed of Li_2_MO_3_ and LiMO_2_ nanoscale domains using electron microscopy (HAADF-STEM). Solid-state NMR further provided a quantitative information about the composite structure (i.e., composition and ratio of the domains), which consists of pure Li_2_MnO_3_, Li_2_[Ni_1/6_Mn_5/6_]O_3_, and Li[Ni,Mn]O_2_ nanoscale domains. We found that Li ions are sequentially and inhomogeneously extracted from the composite structure. Li ions were deintercalated from the Li[Ni,Mn]O_2_ domain in the potential slope region during initial charging, where the Ni ions were redox active for charge compensation. Subsequently, Li ions were preferably extracted from both the Li and TM layers in the Li_2_MnO_3_ domain in the potential plateau region at a high voltage, where the charge was compensated by oxygen anions by formation of ligand holes, peroxide-like moieties, or O_2_ release. Li ions mostly remained in the Li layer of the Li_2_[Ni_1/6_Mn_5/6_]O_3_ domain at 4.8 V. The XRD profiles indicated a reversible phase separation into layered rock-salt and spinel-like nanodomain structures at 4.8 V, where the latter phase is associated with TM ion migration from the TM layer into the vacant Li layer in the Li_2_MnO_3_ domain. During Li intercalation into the structure, cation remigration and mixing formed a domainless layered structure, and the active material became a single Li[Ni_0.25_Mn_~0.75_]O_2_ phase with a small content of remnant Li_2_MnO_3_ domain. After the 1^st^ charge–discharge cycle, when irreversible structural activation occurred, the bulk structure showed reversible Li extraction accompanied by reversible lattice changes and phase separation into layered and spinel-like phases at high potentials. These results provide a new insight into the delithiation/lithiation behavior of nanoscale composite-structured LMRs, which further serve as understanding of the capacity and voltage fading mechanism relating to structural degradation of these materials.

## Experimental methods

Li_1.2_Ni_0.2_Mn_0.6_O_2_ was synthesized via solid-state reaction at 900 °C for 12 h with the starting materials, LiOH · H_2_O, Ni_2_CO_3_ (Wako Pure Chemical Industries) and MnCO_3_ (Kojundo Chemical Laboratory)^42,43^. From scanning electron microscopy (SEM) observations, the average particle size was estimated to be ~5 μm, composed of an aggregate of small crystallites (~200 nm in diameter). The chemical composition of the obtained material was determined to be Li_1.25_Ni_0.20_Mn_0.55_O_2–*δ*_ (*δ* ≈ 0.07) via inductively coupled plasma-atomic emission spectrometry (ICP-AES; ICPS-8100, Shimadzu) and iodometric titration measurement that estimates the average oxidation state of the transition metals.

A positive electrode was prepared using a mixture of the active material, acetylene black (Denki Kagaku Kogyo), and polyvinylidene difluoride (PVDF, Kureha) at a weight ratio of 80:10:10 spread onto an aluminum foil with 1-methyl-2-pyrrolidone (NMP) and dried at 80 °C under vacuum overnight.^43,56^ The electrode was pressed to a thickness of 30–35 μm and foils of metallic lithium (0.2 mm in thickness, >99.9%, Honjo Metal) were used as counter and reference electrodes. These components were assembled with the Celgard 2500 separator and soaked in an electrolyte solution, and sealed in an aluminum-coated laminate-type cell in an Ar-filled glove box. The electrolyte solution used herein was 1 M LiPF_6_ solution dissolved in anhydrous ethylene carbonate (EC) and ethylmethyl carbonate (EMC) at a volumetric ratio of 3:7 (Kishida Chemical).

The electrochemical measurements were performed at 50 °C on an automatic cycling and data recording system (HJ1001SD8, Hokuto Denko). The cells were galvanostatically cycled between 2.0 and 4.8 V vs. Li/Li^+^ at a current rate of 20.5 mA g^-1^ ^56^. The cells were carefully disassembled at the desired charge/discharge states in the glove box and rinsed with dimethyl carbonate (DMC) to remove any electrolyte solution residue.

Synchrotron-radiation XRD (SR-XRD) measurements were performed using powdered samples sealed in 0.5 mm*ϕ* borosilicate glass capillaries in the 2*θ* range of 4.0–52.7° with Δ*θ* of 0.0025° at the BL28XU beamline in SPring-8 (Hyogo, Japan). An incident radiation of *λ* = 0.4997 Å was used, which was calibrated with the lattice parameters of CeO_2_. The Rietveld refinements of the crystal structure parameters were performed using Jana2006^69^.

Soft XAS measurements were performed at beamline BL-11 in the SR Center, Ritsumeikan University (Shiga, Japan). The samples were placed into a sample holder in the glove box and transferred using a transfer vessel to the high vacuum sample chamber without exposure to the air. The Ni, Mn L_II,III_-edge, and O K-edge spectra were acquired in partial fluorescence yield (PFY) mode. Mn L_II,III_-edge spectra were also acquired in inverse partial fluorescence yield (IPFY) mode to account for self-absorption effects^70^. The PFY/IPFY spectra are (relatively) bulk-sensitive with a probing depth of up to ~200 nm^71^.

^7^Li MAS NMR spectra were acquired using an ECA-600 spectrometer (JEOL RESONANCE Inc.) at a magnetic field of 14.1 T with a wide-bore T3 MAS probe (Agilent Technologies). The powder samples were packed into 1.6 mm*ϕ* MAS ZrO_2_ rotors with airtight caps in a glove box and spun at a spinning rate of 30 kHz during the experiments. The practical temperatures of the spinning samples at 30 kHz were estimated to be ~60 °C due to frictional heating. To obtain high resolution spectra, the pj-MATPASS technique^40^ was used with a π/2 pulse width of 1.1 μs and a relaxation delay of 0.1 s. All spectra were referenced to a 1 M LiCl solution at 0.0 ppm. The signal intensities were normalized by the sample weight in the rotors.

## Supplementary information

Supplementary information.
