# Supplementary material for: Sequential delithiation behavior and structural rearrangement of a nanoscale composite-structured Li1.2Ni0.2Mn0.6O2 during charge–discharge cycles
Source: Sci Rep. 2020 Jun 22;10:10048. doi: 10.1038/s41598-020-66411-0 (PMC7308291; doi:10.1038/s41598-020-66411-0)
Supplement: Supplementary file 1 — Supplementary information. [file 41598_2020_66411_MOESM1_ESM.docx]

**Supplementary Information**

**Sequential delithiation behavior and structural rearrangement of a nanoscale composite-structured Li_1.2_Ni_0.2_Mn_0.6_O_2_ during charge–discharge cycles**

Keiji Shimoda^1,^*, Koji Yazawa^2^, Toshiyuki Matsunaga^1^, Miwa Murakami^1^, Keisuke Yamanaka^3^, Toshiaki Ohta^3^, Eiichiro Matsubara^4^, Zempachi Ogumi^1^, Takeshi Abe^5^

^1^ *Office of Society-Academia Collaboration for Innovation, Kyoto University, Uji, Kyoto 611-0011, Japan*

^2^ *JEOL RESONANCE Inc., Akishima, Tokyo 196-8558, Japan*

^3^ *SR Center, Ritsumeikan University, Kusatsu, Shiga 525-8577, Japan*

^4^ *Department of Materials Science and Engineering, Kyoto University, Kyoto 606-8501, Japan*

^5^ *Gradual School of Global Environmental Studies, Kyoto University, Katsura, Nishikyo, Kyoto 615-8510, Japan*


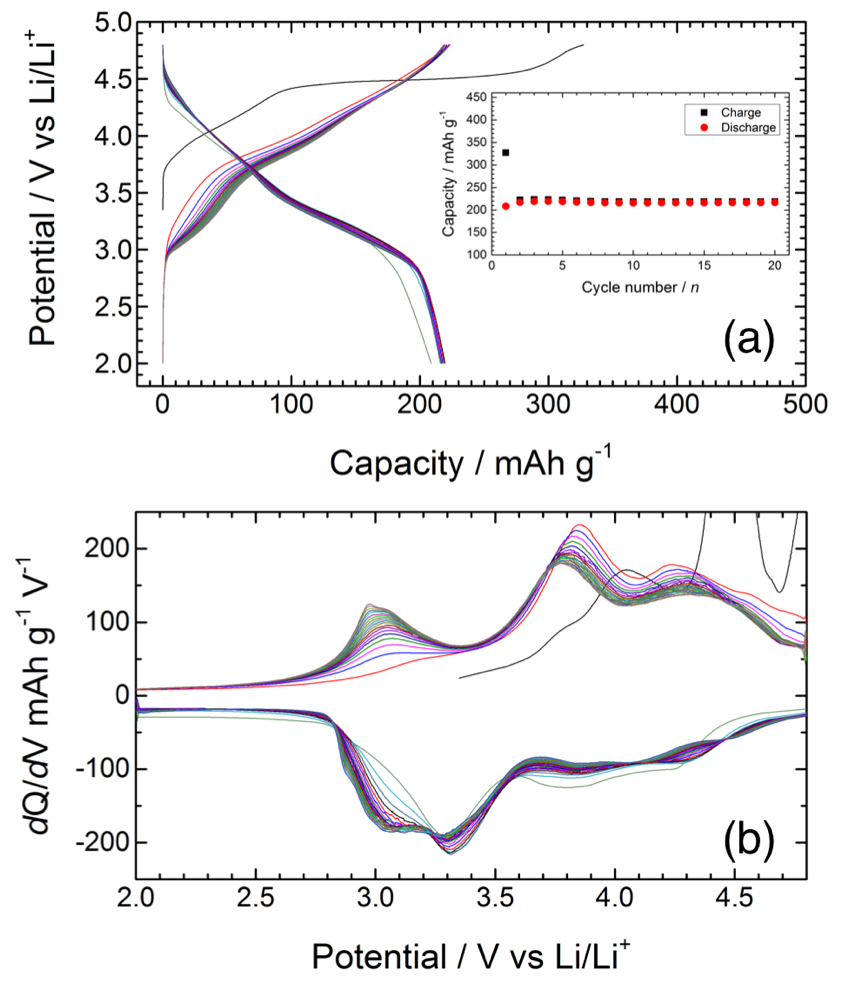


1. (a) Charge–discharge profiles and (b) d*Q*/d*V* curves of the Li//Li_1.2_Ni_0.2_Mn_0.6_O_2_ cell up to the 20^th^ cycle. The electrochemical measurements were performed at 50 °C between 2.0 and 4.8 V vs. Li/Li^+^ with a constant current of 20.5 mA g^–1^.


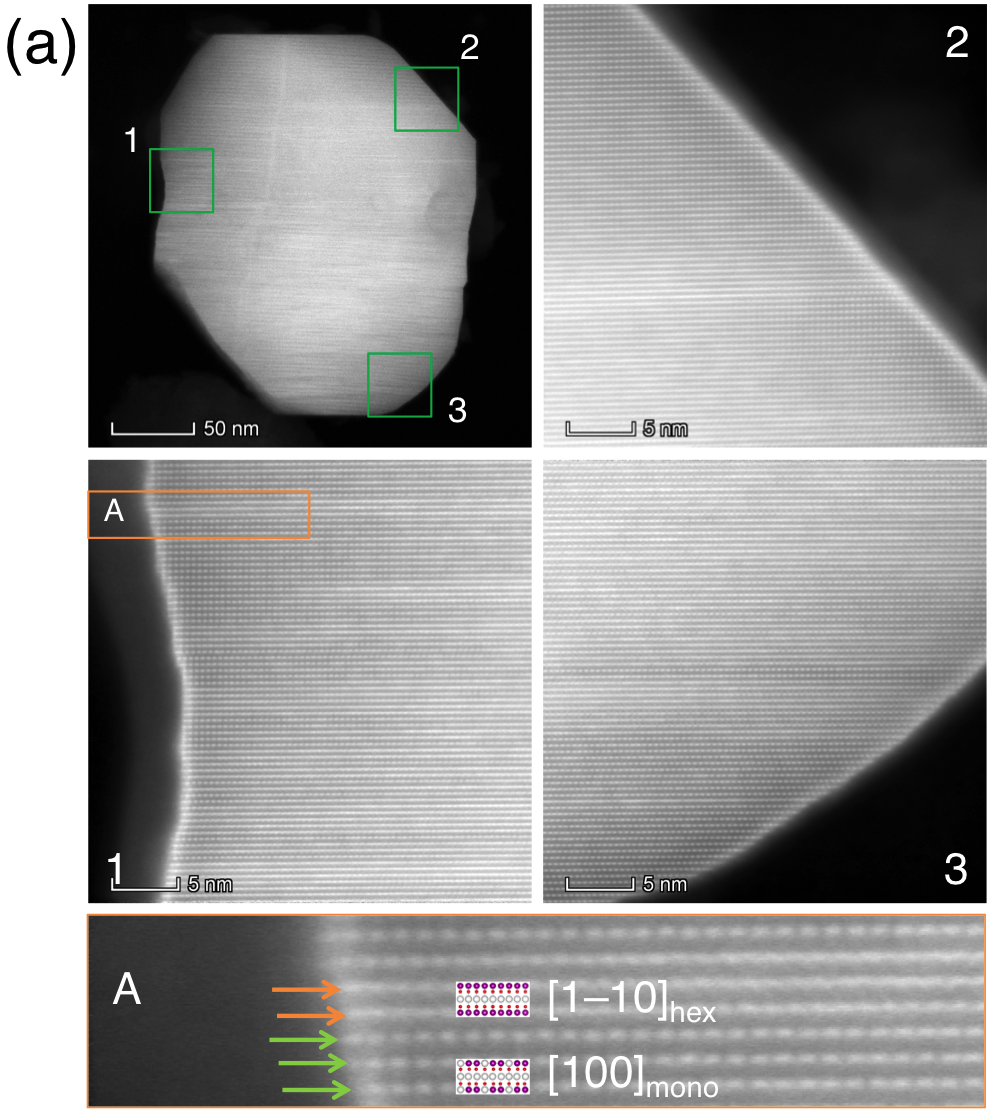


1. (a) HAADF-STEM images of the pristine Li_1.2_Ni_0.2_Mn_0.6_O_2_ sample. (b) EDS mapping images and its intensity profile (gray value) in the rectangular region. The HAADF-STEM images were acquired at an accelerating voltage of 200 kV in the drift corrected frames integration (DCFI) mode using Talos F200X (FEI).


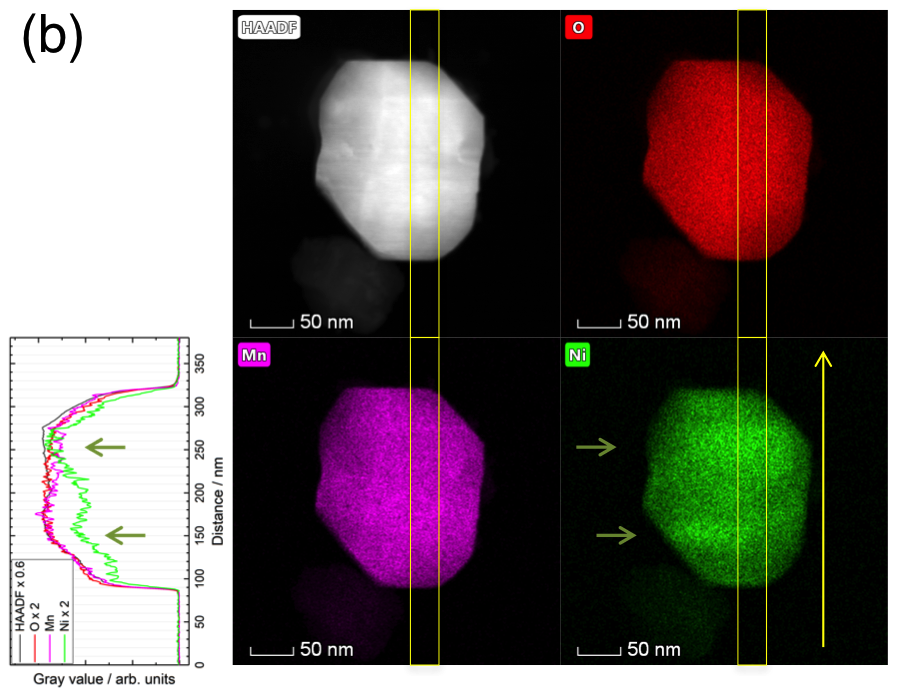


Fig. S2. (continued).


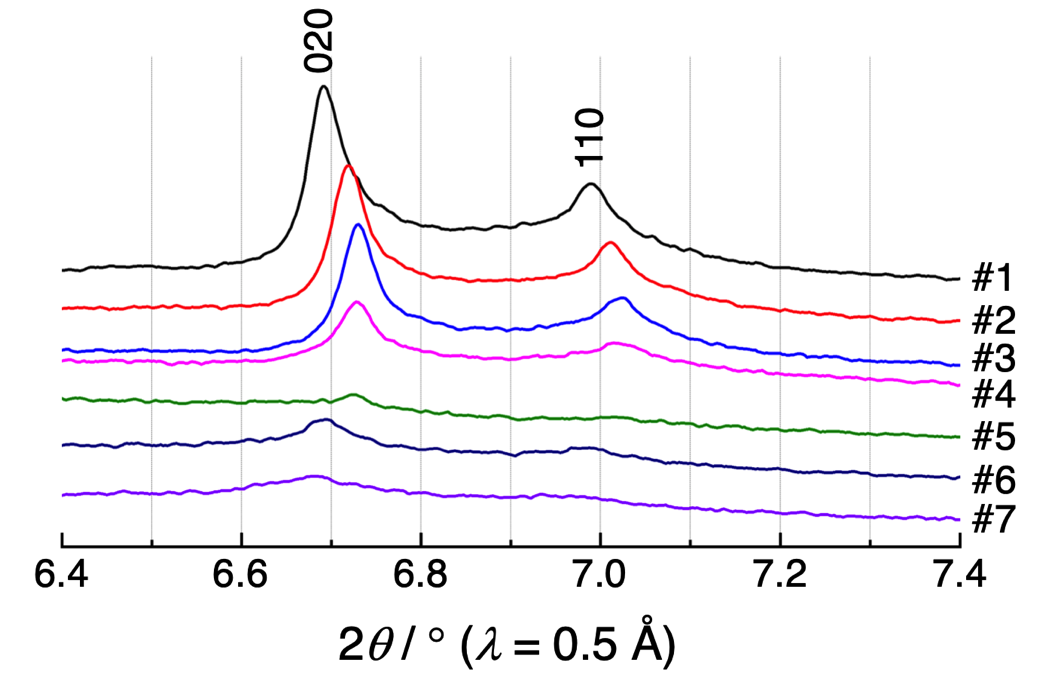


1. SR-XRD profiles of the Li_1.2_Ni_0.2_Mn_0.6_O_2_ electrode at the 1^st^ cycle. The superlattice peaks (020 and 110 reflections) of the *C*2/*m* structure were enlarged. The sample numbers are described in Fig. 1.


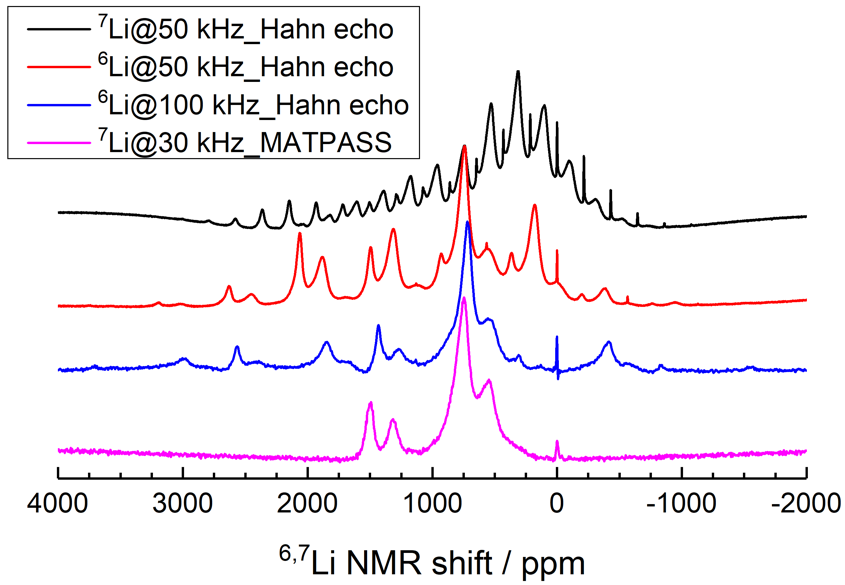


1. Comparison of ^6,7^Li MAS NMR spectra of the pristine Li_1.2_Ni_0.2_Mn_0.6_O_2_ sample.


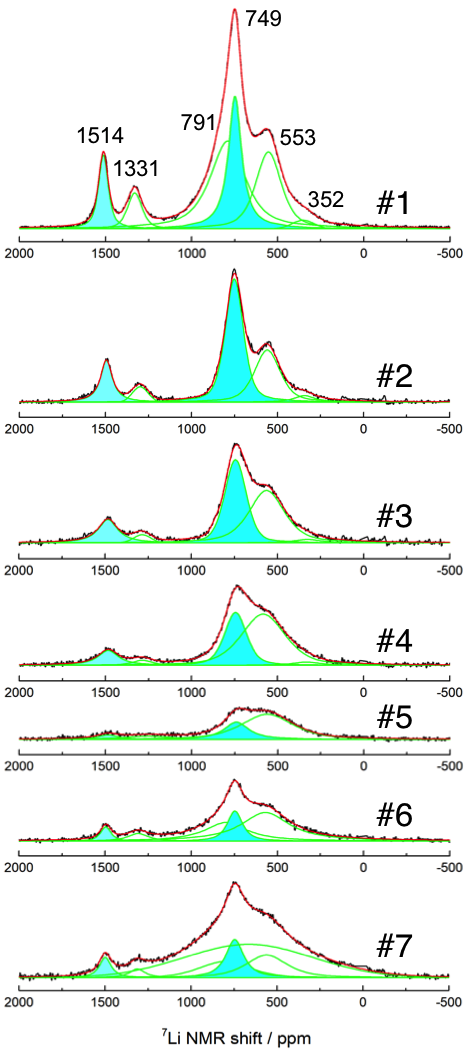


1. Peak deconvolution of the ^7^Li pj-MATPASS spectra of the Li_1.2_Ni_0.2_Mn_0.6_O_2_ electrode at the 1^st^ cycle.


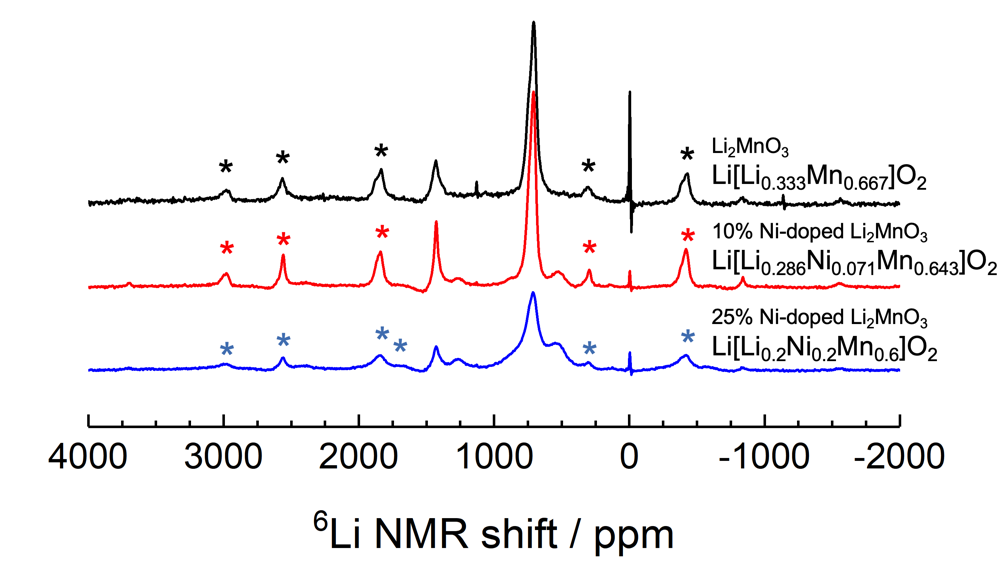


1. ^6^Li MAS NMR spectra of Li_2_MnO_3_ and Ni-doped Li_2_MnO_3_ samples. These spectra were acquired with the spinning rate of 100 kHz. Asterisks indicate spinning sidebands.


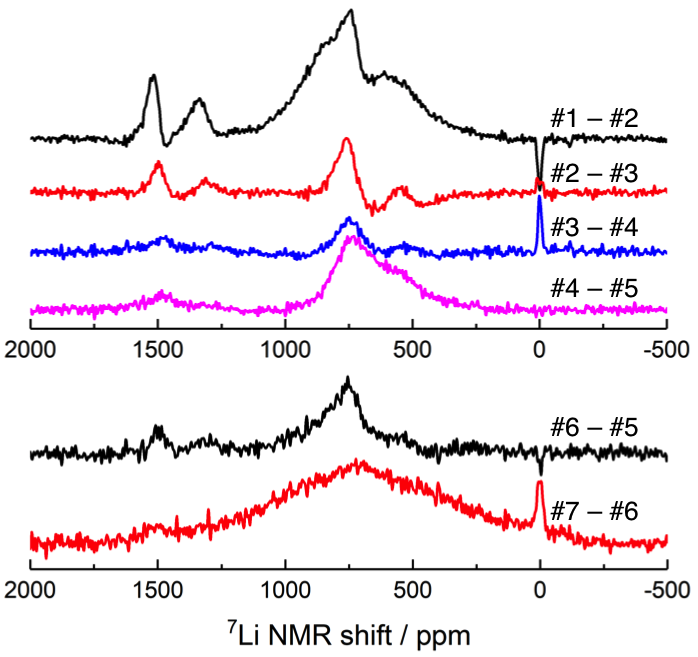


1. Difference pj-MATPASS difference spectra of the Li_1.2_Ni_0.2_Mn_0.6_O_2_ electrode at the 1^st^ cycle..


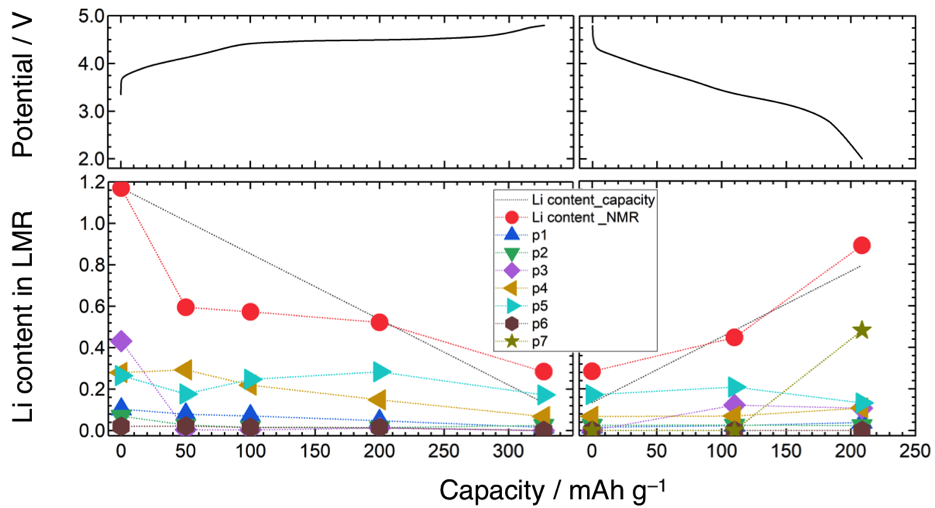


1. Evolution of each ^7^Li signal component in Fig. S5 during the 1^st^ cycle. Component p1: 1514 ppm, p2: 1331 ppm, p3: 791 ppm, p4: 749 ppm, p5: 553 ppm, p6: 352 ppm, and p7: 666 ppm (a new component in #7).


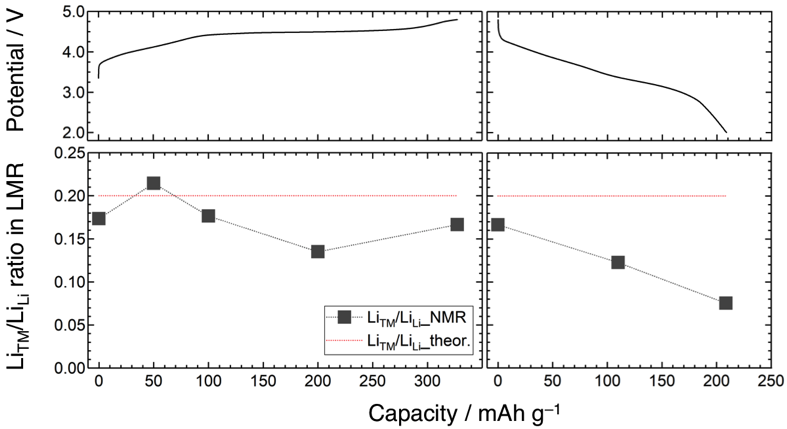


1. Evolution of the Li_TM_/Li_Li_ ratio in the Li_1.2_Ni_0.2_Mn_0.6_O_2_ electrode during the 1^st^ cycle.


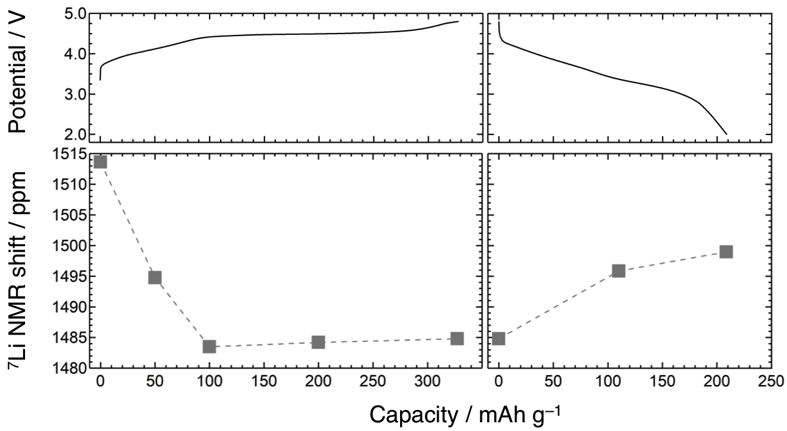


1. Evolution of the 1514 ppm peak position during the 1^st^ cycle.

Table S1. The average Ni and Mn valence states estimated from the soft XAS.

|  | Sampling point | Ratio of Ni^2+^ | Ratio of Ni^4+^ | Average Ni valence |
| --- | --- | --- | --- | --- |
| 1^st^ cycle | #1 | 0.53 | 0.47 | 2.9 *^b^* |
|  | #3 | 0 | 1 *^a^* | 4 |
|  | #4 | 0.21 | 0.79 | 3.6 |
|  | #5 | 0.50 | 0.50 | 3.0 |
|  | #7 | 1 *^a^* | 0 | 2 |
| 2^nd^ cycle | #9 | 0.52 | 0.48 | 3.0 |
|  | #10 | 1 | 0 | 2 |
| 20^th^ cycle | #11 | 0.72 | 0.28 | 2.6 |
|  | #12 | 1 | 0 | 2 |

|  | Sampling point | Ratio of Mn^2+^ *^c^* | Ratio of Mn^3+^ *^c^* | Ratio of Mn^4+^ *^c^* | Average Mn valence |
| --- | --- | --- | --- | --- | --- |
| 1^st^ cycle | #1 | 0.0 | 0.03 | 0.97 | 4.0 |
|  | #3 | 0.0 | 0.08 | 0.93 | 3.9 |
|  | #4 | 0.0 | 0.12 | 0.88 | 3.9 |
|  | #5 | 0.0 | 0.15 | 0.85 | 3.8 |
|  | #7 | 0.0 | 0.51 | 0.49 | 3.5 |
| 2^nd^ cycle | #9 | 0.0 | 0.14 | 0.86 | 3.9 |
|  | #10 | 0.0 | 0.63 | 0.37 | 3.4 |
| 20^th^ cycle | #11 | 0.0 | 0.29 | 0.71 | 3.7 |
|  | #12 | 0.0 | 0.74 | 0.26 | 3.3 |

*^a^* For the linear combination fitting of the Ni L_III_-edge spectra, it was assumed that the spectra #3 and #7 are representative of Ni^4+^ and Ni^2+^ states, respectively.

*^b^* The average Ni valence for the pristine sample seems to be overestimated probably due to larger contributions from the particle surface and/or incomplete assumption described above.

*^c^* For the linear combination fitting of the Mn L_III_-edge spectra, the reference spectra of MnO (Mn^2+^), Mn_2_O_3_ (Mn^3+^), and Li_2_MnO_3_ (Mn^4+^) were used.

**Determination of chemical composition and ratio of the nanodomains**

The chemical compositions of the three phases were determined as follows: The Li_2_MnO_3_ domain was clearly identified from 749 and 1514 ppm signals in the ^7^Li MAS NMR spectrum. The Li_TM_/Li_Li_ intensity ratio was 0.37 (ideally 0.33). Therefore, the calculated composition is expressed as Li_1.37_Mn^4+^_0.63_O_2(–_*_δ_*_)_, where oxygen vacancy may be introduced for charge neutrality. Similarly, Li_2_[Ni_1/6_Mn_5/6_]O_3_-like domain was identified from 553 and 1331 ppm signals. The Li_TM_/Li_Li_ ratio was 0.26, and the Ni/Mn ratio was fixed at 1/5, and the calculated composition is expressed as Li_1.26_Ni^4+^_0.11_Mn^4+^_0.57_O_2_, assuming that the Ni valence is 4+ in Li_2_MO_3_. We know the Li content in each domain from ^7^Li MAS NMR spectrum; Li_1.37_Mn_0.63_O_2_ : Li_1.26_Ni_0.11_Mn_0.57_O_2_ : LiMO_2_ = 0.33 : 0.30 : 0.37. This should be rescaled as Li_1.37_Mn_0.63_O_2_ : Li_1.26_Ni_0.11_Mn_0.57_O_2_ : LiMO_2_ = 0.28 : 0.28 : 0.43 (normalized to O). Then, the remaining LiMO_2_ phase composition can be calculated by subtracting 0.28Li_1.37_Mn_0.63_O_2_ and 0.28Li_1.26_Ni_0.11_Mn_0.57_O_2_ from the bulk composition Li_1.17_Ni_0.21_Mn_0.59_O_2_, that is, 0.43LiNi_0.41_Mn_0.57_O_2_.
